# Ten simple rules for making training materials FAIR

**DOI:** 10.1371/journal.pcbi.1007854

**Published:** 2020-05-21

**Authors:** Leyla Garcia, Bérénice Batut, Melissa L. Burke, Mateusz Kuzak, Fotis Psomopoulos, Ricardo Arcila, Teresa K. Attwood, Niall Beard, Denise Carvalho-Silva, Alexandros C. Dimopoulos, Victoria Dominguez del Angel, Michel Dumontier, Kim T. Gurwitz, Roland Krause, Peter McQuilton, Loredana Le Pera, Sarah L. Morgan, Päivi Rauste, Allegra Via, Pascal Kahlem, Gabriella Rustici, Celia W. G. van Gelder, Patricia M. Palagi

**Affiliations:** 1 ZB MED Information Centre for Life Sciences, Cologne, Germany; 2 Bioinformatics group, Department of Computer Science, University of Freiburg, Freiburg, Germany; 3 European Molecular Biology Laboratory, European Bioinformatics Institute (EMBL-EBI), Wellcome Genome Campus, Hinxton, Cambridgeshire, United Kingdom; 4 Netherlands eScience Center, Amsterdam, the Netherlands; 5 Dutch Techcentre for Life Sciences, Utrecht, the Netherlands; 6 Institute of Applied Biosciences, Centre for Research and Technology Hellas, Thessaloniki, Greece; 7 Department of Computer Science, The University of Manchester, Manchester, United Kingdom; 8 Open Targets, Wellcome Genome Campus, Hinxton, United Kingdom; 9 Biomedical Sciences Research Center "Alexander Fleming”, Vari, Greece; 10 Institut Français de Bioinformatique, Génoscope, France; 11 Institute of Data Science, Maastricht University, Maastricht, the Netherlands; 12 University of Cambridge, Cambridge, United Kingdom; 13 University of Luxembourg, Esch-sur-Alzette, Luxembourg; 14 Oxford e-Research Centre, Department of Engineering Sciences, University of Oxford, United Kingdom; 15 IBIOM-CNR, Bari, Italy; 16 IBPM-CNR, Sapienza Università di Roma, Roma, Italy; 17 CSC—IT Center for Science, Keilaranta, Espoo, Finland; 18 Scientific Network Management S.L., Barcelona, Spain; 19 SIB Training group, SIB Swiss Institute of Bioinformatics, Lausanne, Switzerland; Dassault Systemes BIOVIA, UNITED STATES

## Abstract

Everything we do today is becoming more and more reliant on the use of computers. The field of biology is no exception; but most biologists receive little or no formal preparation for the increasingly computational aspects of their discipline. In consequence, informal training courses are often needed to plug the gaps; and the demand for such training is growing worldwide. To meet this demand, some training programs are being expanded, and new ones are being developed. Key to both scenarios is the creation of new course materials. Rather than starting from scratch, however, it’s sometimes possible to repurpose materials that already exist. Yet finding suitable materials online can be difficult: They’re often widely scattered across the internet or hidden in their home institutions, with no systematic way to find them. This is a common problem for all digital objects. The scientific community has attempted to address this issue by developing a set of rules (which have been called the Findable, Accessible, Interoperable and Reusable [FAIR] principles) to make such objects more findable and reusable. Here, we show how to apply these rules to help make training materials easier to find, (re)use, and adapt, for the benefit of all.

## Introduction

Worldwide demand for bioinformatics and computational biology training continues to grow. This demand has been met by increasing the supply of training opportunities, including face-to-face workshops [1], e-learning [2], webinars [3], etc. A major task in developing new training sessions is preparing training materials, which can be time consuming and challenging for both new and experienced trainers. One solution is to find and (re)use existing materials. This requires that they have been shared, properly described, and made available for (re)use by their authors; but finding suitable online materials that aren’t subject to licensing and/or copyright restrictions can be hard. They are also often scattered across different repositories, are siloed in their home institutions, or lack the metadata required to enable their (re)use. If we are to meet the demand for bioinformatics and computational biology training, we need to share and deliver training materials consistently, following best practices that enable their (re)use and adaptation [4,5].

Having encountered similar challenges with other digital objects [6], the scientific research community published the FAIR principles [7] and guidelines on how to apply them (e.g., GO FAIR [8] and the Association of European Research Libraries (LIBER) [9]. Within the European life sciences infrastructure for biological information (ELIXIR) [10], the Training Platform [11] is collecting and sharing information about training materials from 23 participating European nodes via the Training e-Support System (TeSS) [12]. As part of that process, we are exploring the application of the FAIR principles to those materials, in order to improve their (re)usability.

**Fig 1:**
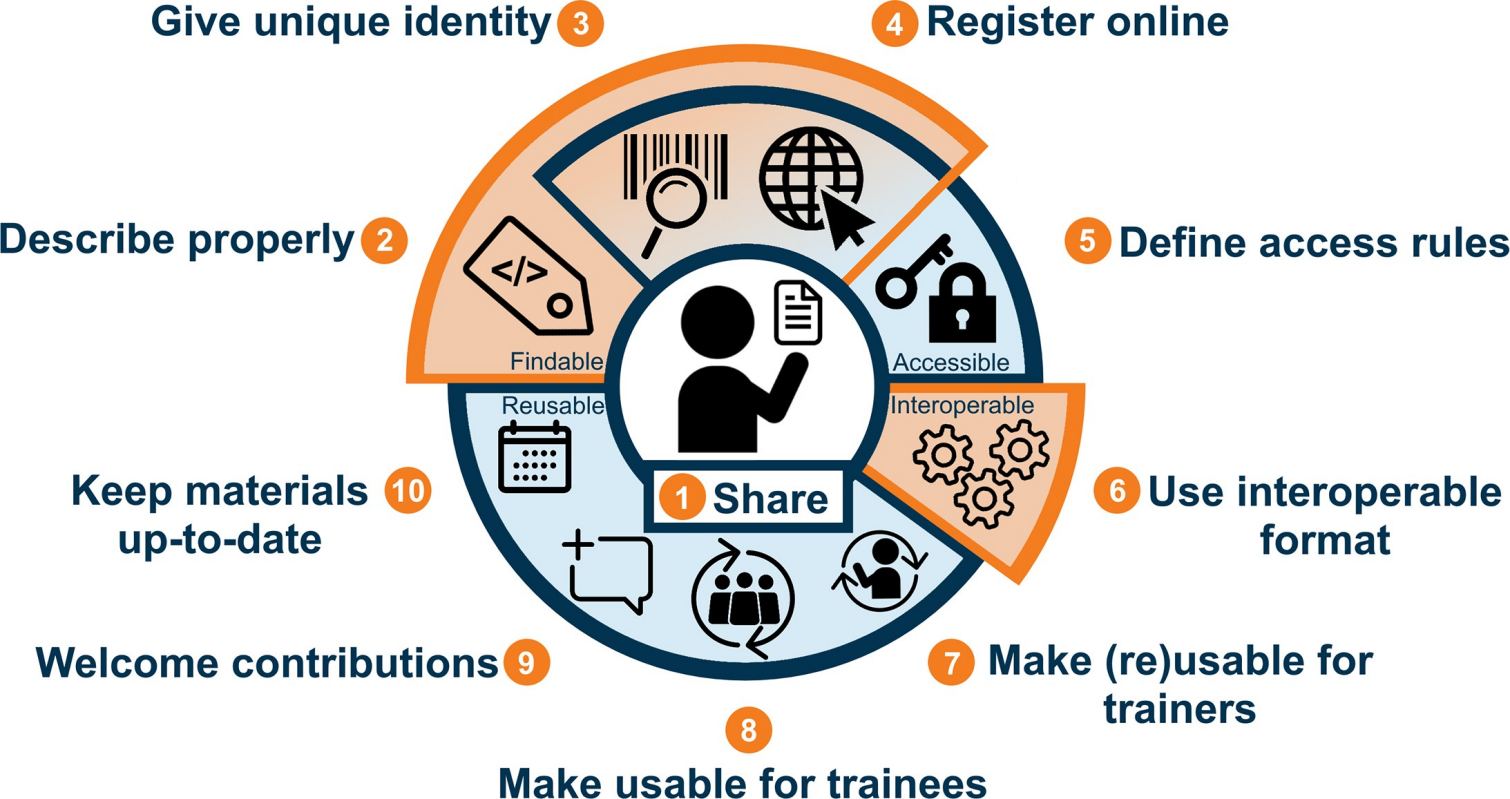
Ten simple rules for making training materials FAIR. The first rule—to share—is the central starting point; the Findability rules include description, identity, and registration; the latter two, together with access rules, correspond to Accessibility; Interoperability stands on its own, with one rule about formats; the remaining four rules cover different aspects of Reusability. Illustration from Luc Wiegers and Celia van Gelder: https://doi.org/10.5281/zenodo.3593257.

Here, we offer trainers some simple rules, summarized in Fig 1, to help make their training materials FAIR, enabling others to find, (re)use, and adapt them. We use a broad definition of “training materials” to include any digital object used in a training context (e.g., slide presentations, exercises, datasets, etc.).

## Rule 1: Plan to share your training materials online

As a trainer, you are likely to be a passionate teacher and keen to share your expertise. Sharing your materials is one way to achieve this, a simple step that can bring many benefits:

- For you, it provides a record (and recognition) of the training that you have developed
- For other trainers, it can provide inspiration, in terms of the content covered and method of delivery
- For trainees, it provides a navigable landscape in which to find relevant training resources and build personalized learning paths
- For the bioinformatics community, it facilitates systematic training-gap analyses and development of additional materials and courses.

There are many ways to share materials: These include personal or institutional Web servers; cloud-based collaboration tools (e.g., Google Drive and Dropbox); cross-domain repositories (e.g., figshare [13], GitHub [14], and YouTube [15]); or specialized data repositories for datasets that are integral to particular training sessions.

Ultimately, how and where you share your training materials depends on your individual circumstances (including institutional rules). Various guides have been developed to help choose suitable data repositories [16,17], and much of the advice they offer also applies to choosing where to share training materials. It is better to share than not to share; however, before you do, it is important to reflect on how you can make your materials FAIR and how your choice of repository may affect this. Doing this from the outset will allow you to disseminate your work more efficiently and more widely.

## Rule 2: Improve findability of your training materials by properly describing them

Describing digital objects with structured metadata is fundamental to making them FAIR. Regardless of the type of object, adding appropriate, standardized metadata will help make them both machine and human readable. Metadata can be hosted outside the digital object itself, boosting findability and preserving information, even when the digital object has ceased to exist.

Schema.org [18] is a collaborative project that facilitates the addition of structured metadata to web pages. It describes data types (movies, books, etc.) and properties (actor, author, etc.), which can be indexed by search engines and used to provide snippets of information (like those that appear in Google searches). BioSchemas [19], a community initiative to extend the Schema.org standard to life-science resources, includes a specification for TrainingMaterial [20]. This specification, alongside those for Course and CourseInstance, constitutes a joint effort between the ELIXIR Training Platform and Global Organisation for Bioinformatics Learning, Education and Training (GOBLET) [21,22], providing community-endorsed metadata standards to make training materials more discoverable. The specification offers guidance on the kind of information to provide when sharing your training resources (e.g., prerequisites, target audience, and learning outcomes). Once annotated using BioSchemas specifications, training resources can be automatically aggregated by training registries like TeSS.

Describing and annotating training materials with relevant keywords from controlled vocabularies, taxonomies, thesauruses, or ontologies is also important. This reduces ambiguity and facilitates discovery and retrieval of information by improving the efficacy of metadata filtering. An example of ontology is EDAM [23], which includes terms for data types, data identifiers, data formats, operations, and topics. Other ontologies can be found in resources like FAIRsharing [24], the Ontology Lookup Service [25] or BioPortal [26].

## Rule 3: Give your training materials a unique identity

Training materials, like any digital object whose unique identifier is just a uniform resource locator (URL), are at risk of disappearing, [27] because of service retirement, link instability (e.g., when web domains are no longer available), or other factors. This problem, also known as “link rot” [28], can be addressed by using persistent identifiers (PIDs). A PID is a unique identification code that is attached to a digital object and registered at an agreed location. It is guaranteed to remain functional, even if an organization’s URL changes [29].

Providing PIDs for training materials makes them easier to cite and helps research-metric systems to count those citations. The most commonly used PID systems are the persistent uniform resource locator (PURL) [30], the Handle System [31], the Archival Resource Key (ARK) [32], and the digital object identifier (DOI) [33].

Other PIDs play important roles in the identification of training materials, datasets, software, and other digital objects. For instance, Open Researcher and Contributor ID (ORCID) [34] focuses on researcher identification, making it easier for authors to receive credit for their work, regardless of variations in their names. To increase the FAIRness of training materials and the recognition of their authors, such PIDs can be added to a citation file or README file and/or section that accompanies the requisite metadata. Although PIDs are not sufficient to guarantee FAIRness, they grant some level of persistence and integrity.

## Rule 4: Register your training materials online

To make your materials more discoverable, it is helpful to share them via an online registry that targets a specific audience (e.g., bioinformatics, physics, etc.). ELIXIR's training portal, TeSS, is a centralized metadata registry that allows browsing and discovery of life-science training events and materials currently dispersed across the internet. TeSS allows users to register content manually, which carries a significant overhead, requiring providers to ensure that they add their resources in a timely way and update them regularly. The preferred, and hence primary, mechanism for collecting training metadata in TeSS is to aggregate data from content providers automatically. This involves the use of bespoke “scrapers” that extract information from a variety of trusted websites [35].

The GOBLET training portal [36] also allows manual upload of training events and materials. As in TeSS, resources are tagged using terms from the EDAM ontology and must be kept up to date by content providers. However, in addition, the GOBLET portal imports and displays training information from TeSS via an embedded widget. This means that GOBLET is both a content provider and a vehicle to disseminate training information harvested by TeSS. Other registries include open educational resources (OER) commons [37].

## Rule 5: Define access rules for your training materials

Accessibility refers to the ability to retrieve content. Access to training materials may be open or limited via an access-request mechanism. Authentication may be required owing to membership (i.e., a website’s content may be limited to members), restricted domains (e.g., those available only for students in a particular university), or paid options (i.e., content is only available for a fee). Whether your training materials are open or restricted should be clearly stated as part of their metadata. It is also advisable to state the accessibility rules in plain English (e.g., on the website hosting your materials) so that others know how to get access. If you collect usage data, it is important to include data-protection information, either as part of the material documentation, the hosting website disclaimers, or the material’s metadata.

## Rule 6: Use an interoperable format for your training materials

Training materials need to be captured in interoperable formats, so that they can be used in different contexts (e.g., operating systems and software) and built upon later.

For materials like slides, it is important that other trainers are able to (re)use, fine-tune or even extend them. This means that you should choose a format that supports editing and extension. Here, the de facto standard is Microsoft PowerPoint [38], which is only available for computers running Windows or macOS. The default file format used by Microsoft PowerPoint is the Open XML Presentation (PPTX), which is compatible with other open-source alternatives, such as Apache OpenOffice Impress [39] and LibreOffice Impress [40].

Other proprietary tools, like Keynote [41], use very limiting software-specific file formats.

Another commonly used open file format is the Portable Document Format (PDF) [42], which is compatible with a variety of different operating systems, browsers, software, etc. However, PDF documents are not easy to edit and can therefore be difficult to (re)use. An alternative is to use a LaTeX [43] class (e.g., Beamer (41)), which takes a structured source file, compiles it, and then outputs a PDF file. Tools like this, however, require you to have the requisite technical skills. If you do use PDFs, we recommend that you also make the files of origin (whether PPTX or LaTeX) available, so that other trainers can modify and adapt them to their needs.

There is also a growing trend to provide training materials in Markdown (MD) and reStructuredText (RST) format and to make them available via services like Read the Docs [44] and GitHub. These formats present low barriers to learning and allow (re)usability and version control. However, they work best when available as online resources, which may be limiting in settings with low connectivity.

Materials such as hands-on exercises may include related software and data, which should also be provided in interoperable formats, following the FAIR principles [7,45]. An overview of the main advantages and disadvantages of the most common training material formats is provided in Table 1.

**Table 1:** Comparison of common training material formats.

Format	Advantages	Disadvantages
PPT and PPTX	• Easily (re)usable • Available to multiple OSs/Software • Widespread	• Limited way to provide detailed training instructions • Not version controlled
Keynote	• Polished overall aesthetic	• Limited to macOS family • Not version controlled
PDF	• Can be displayed identically in any environment	• Not easily editable • Not version controlled
TeX	• Easily editable • Version controlled • Free	• Steep learning curve for trainers
MD, RST, and HTML	• Version controlled Free	• Rendering (need templating to transform into HTML)
Google slides	• Version controlled Free	• Not always possible to use owing to local/institutional policies • Not always accessible (depending on geographic location)

MD, Markdown; PDF, Portable Document Format; PPT, PowerPoint; PPTX, PowerPoint Open XML Presentation; RST, reStructuredText

## Rule 7: Make your training materials (re)usable for trainers

You may wish to (re)use someone else’s materials in whole, in part or just for inspiration. Regardless, this will require those materials to be updated and adapted to new contexts to consider, for example, different audiences or changes in the field. Training materials can be made easier for others to (re)use and adapt by applying an appropriate license and annotating them with metadata (see Rule 2).

Choosing a license is important. By default, training materials are generally copyrighted in restrictive ways, such that only the original authors and contributors can use, modify, and create derivative works or distribute them. Creative Commons (CC) licenses can be applied to give authors and users appropriate rights of (re)use [46]. It is important to state clearly which license has been chosen and to include information on how the material can be cited, as part of the material’s metadata.

Metadata shared alongside training materials should provide context and sufficient detail to enable others to assess whether the materials are appropriate and adaptable to their own settings. Table 2 provides general guidance on the type of information to include.

**Table 2:** Suggested metadata for training materials.

Type of metadata	What to include
Title	Title of the training material.
Contact details	Author(s) name and contact details.
Licensing and (re)use details	License under which the materials are shared, and rules and conditions for (re)use and contribution.
Preferred citation	Instructions on how to cite your material.
Description	Overview of the subject matter, aims of the training, and language in which the training is delivered.
Learning outcomes	Statements that indicate what trainees should be able to do upon successful completion of the training.
Target audience	The intended audience, their prerequisite knowledge and skills, their general background, and how the training material will help them.
Required resources	Technical resources and related materials (software requirements, datasets, infrastructure requirements, etc.).
Keyword	Keywords or tags identifying the topic of the materials.
Structure and duration	Description of the structure of the materials and setting in which to deliver them, including the time allocated to each part (lectures, exercises, etc.)
Additional information	Items that provide additional information about (re)use and delivery of the materials (e.g., general tips and guidance).
Links and references	Links and references that are relevant to the content but not required for delivery of the materials.
Date of last revision	Date of last update of the materials and the version.

## Rule 8: Make your training materials usable for trainees

Most metadata suggested in Table 2 will also help trainees to identify the most appropriate training resources for their needs. Learning outcomes and prerequisites are particularly informative metadata. For learning outcomes to be useful, they should be formulated using active verbs that express the expected behaviors of trainees, and the knowledge, skills, and abilities they will have acquired. Using a structured approach to articulate prerequisites, target audience, and learning outcomes helps to clarify which trainees will benefit most from the training, the skills they should possess before enrolling on a course or working through a set of materials, and what they can expect to be able to do upon successful completion of their training.

## Rule 9: Make your training materials contribution friendly

If you (re)use training materials, you may wish to provide feedback on the content (e.g., by reporting errors, adding examples, or suggesting alternative explanations). Rules for participation and contribution should be stated. CONTRIBUTING files [47] (which define the rules for contribution) are one way of doing this; they also provide opportunities to share expectations about contributions, contact information, and so on.

Such files are recognized in open source communities and are interoperable with some repositories: e.g., GitHub can display them in issue- or pull-requests. Using a warm tone and suggesting potential initial contributions can help to encourage newcomers to participate, especially if contributions are recognized. All contributors should be listed and thanked in the acknowledgements; the most relevant should be credited as authors. Just as credit should be given to contributors, all (re)used materials should also be acknowledged.

## Rule 10: Keep your training materials up-to-date

It is important to update your training materials and to keep abreast of current trends, new features, or developments in the field (new databases releases, software versions, etc.). When and how often to update your materials will depend on how frequently the resources or computational methods they describe change, whether new exercises or supporting media can be found to add a hint of freshness, and so on. If using screenshots to illustrate particular resources, they should reflect the current versions; similarly, exercises and answers should still work with current releases. Ideally, updated materials should be timestamped, given new PIDs, and added to a specialized online registry; for completeness, old versions should also be archived. If you no longer plan to update your materials, provide the timestamp of the last update. Adopting FAIR principles in your training materials will facilitate future updates by the community and help bring the latest developments to users; it might even inspire other trainers to change and adapt your materials to new audiences and new contexts.

## Final words

We are witnessing an increase in demand and supply of bioinformatics and computational biology training, and this is likely to continue in the years ahead. It is paramount that we make concerted efforts to render training materials FAIR so that everyone can benefit. The road to FAIR training may require us to change how we think; nevertheless, the FAIRification of training materials is an important step towards the democratization of knowledge. We hope that these simple rules will ignite a conversation and collaboration within global training communities. Let these be the first steps towards a paradigm shift in providing FAIR training and education for current and future generations.
